# The heterogeneous CTV-PTV margins should be given for different parts of tumors during tomotherapy

**DOI:** 10.18632/oncotarget.21631

**Published:** 2017-10-06

**Authors:** Ying Tong, Guanzhong Gong, Jinhu Chen, Jie Lu, Tonghai Liu, Pinjing Cheng, Yong Yin

**Affiliations:** ^1^ Radiation Physics Department of Shandong Cancer Hospital Affiliated to Shandong University, Jinan, China; ^2^ School of Nuclear Science and Technology, University of South China, Hengyang, China

**Keywords:** tomotherapy, MVCT, setup errors, CTV-PTV margins, heterogeneity

## Abstract

**Objective:**

The purpose of this study was to evaluate CTV-PTV margins of tumors for tomotherapy.

**Methods:**

Setup errors were analyzed for 151 patients receiving helical tomotherapy treatment. 53 patients had head and neck tumors, 45 had thoracic tumors, 20 had abdominal tumors, and 33 had pelvic tumors. The setup errors were calculated in six directions, i.e. +X (left), -X (right), +Y (head), -Y (foot), +Z (ventral), and -Z (dorsal), after Megavoltage CT (MVCT) images were registered to simulation CT images. And then the CTV-PTV margins were calculated.

**Results:**

The setup errors along the +Z direction were significantly higher than that along the –Z direction (*p*<0.05). The CTV-PTV margins on +X, -X, +Y, -Y, +Z, and -Z directions were asymmetric for all tumors, and the heterogeneity were more remarkable on the +Z and –Z directions. The CTV-PTV margins on +Z and –Z were 4.1 mm, 4.6 mm, 5.2 mm, and 8.4 mm; and 3.9 mm, 7.7 mm, 3.3 mm, and 7.7 mm for head and neck tumors, thoracic tumors, abdominal tumors, and pelvic tumors, respectively.

**Conclusions:**

The CTV-PTV margins for patients with different types of tumors were heterogeneous during tomotherapy. The individual margins of six directions should be given for those patients who accept tomotherapy.

## INTRODUCTION

Tomotherapy is a common practice for optimal dose distribution in precise radiotherapy of tumors, which has the functionalities of intensity modulated radiation therapy (IMRT), image guide radiation therapy (IGRT), and adaptive radiation therapy (ART) [1]. Since tomotherapy has a preferable intensity modulation ability compared to traditional IMRT techniques, the dose gradients can be sharper between tumors and adjacent normal tissue. Thus, tomotherapy can provide higher doses to tumors while reducing the dose of organs at risks (OAR), and then it can achieve a better therapeutic ratio [2].

In addition, tomotherapy with its high-dose gradients has advantages in treating complex-shaped tumors [3]. A previous study showed that helical tomotherapy can reduce the dose to the rectum during prostatic tumors treatment [4]. Another study also indicated that the helical tomotherapy treatment could achieve a lower dose to the adjacent tissues compared with IMRT in breast cancers, and the V_95_ could increase by 21% [5]. Tomotherapy had become the preferred radiation therapy approach for craniospinal irradiation (CSI), as previous studies have demonstrated that tomotherapy can achieve high conformity, excellent dose homogeneity, and healthy tissues paring in CSI [6, 7].

Since irreversible dose deviation would be brought due to uncertainty resulting from the tumors’ target and actual radiative treatment process, tomotherapy cannot accept any errors that could affect radiation dose delivery. Thus, the precise target position and contour is extremely important in tomotherapy.

IGRT can monitor whether tumors receive adequate radiotherapy exposure, which is one of the critical points in tomotherapy. Megavoltage CT (MVCT) is a unique imaging method used in tomotherapy. Despite the low contrast-to-noise ratio, it has similar uniformity and spatial resolutions as diagnostic CT images [8]. In addition, MVCT images can not only verify the position of the patients, they can also delineate the anatomy of the patients to a certain extent [2, 9]. Using MVCT images, the setup errors can be calculated by rigid registering the MVCT images to the CT images. Subsequently, the appropriate margins from clinical target volume (CTV) to planning target volume (PTV) can be obtained for optimal treatment.

According to ICRU Report 62 and 83, the PTV should generally be given priority over CTV by analyzing setup errors to ensure the PTV receives an adequate irradiation dose [10, 11]. In addition, CTV-PTV margins should be large enough to prevent geographical misses of tumors without compromising the effects of the treatment [12]. Since the principle and equipment of tomotherapy is significantly different from the general linear accelerator, it is necessary for tomotherapy to set a specific standard based on its characteristics.

The margins of PTV are traditionally calculated based on setup errors, with most studies calculating the CTV-PTV margins using X, Y, Z directions [13-17]. However, in our recent study, we found that the setup errors in different directions of the same axes maintain heterogeneity. In this study, we analyzed the setup errors on the six axes of all tumors, and investigated the heterogeneity of CTV-PTV margins.

## RESULTS

### The distributions of setup errors

**Figure 1:**
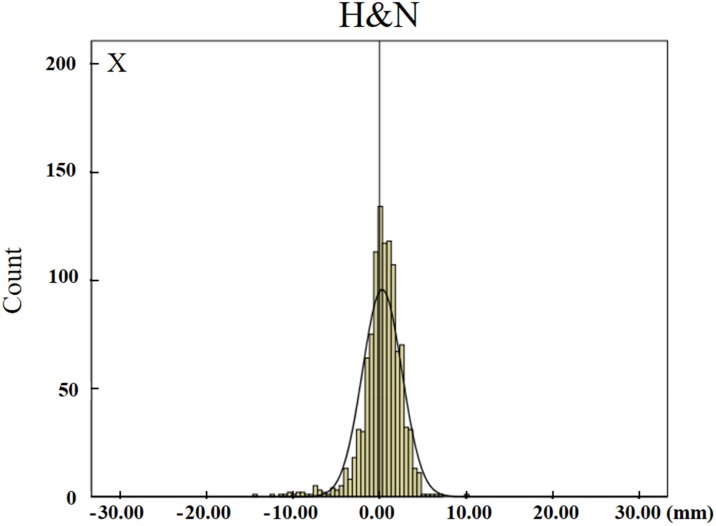

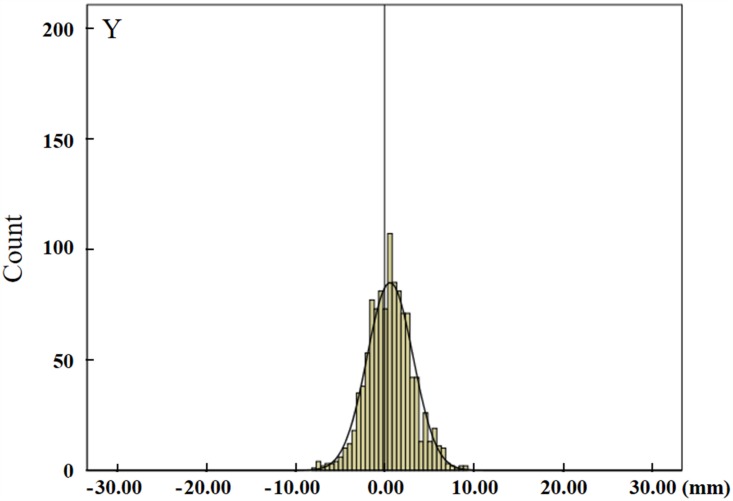

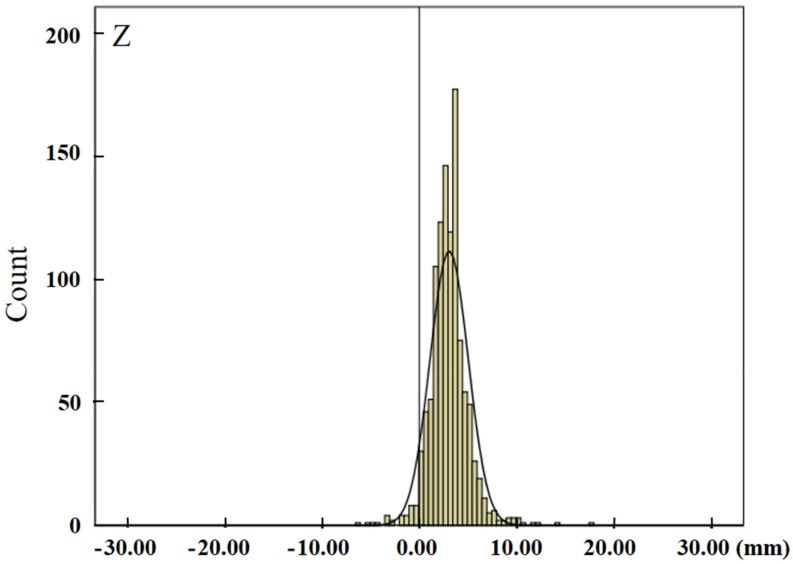

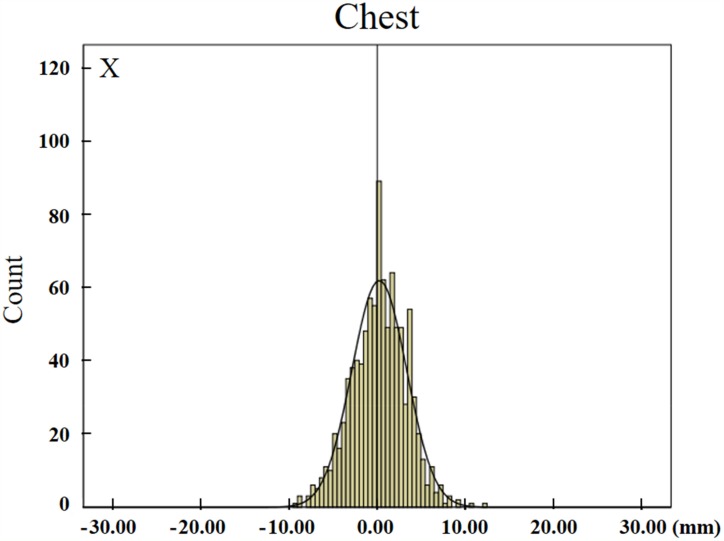

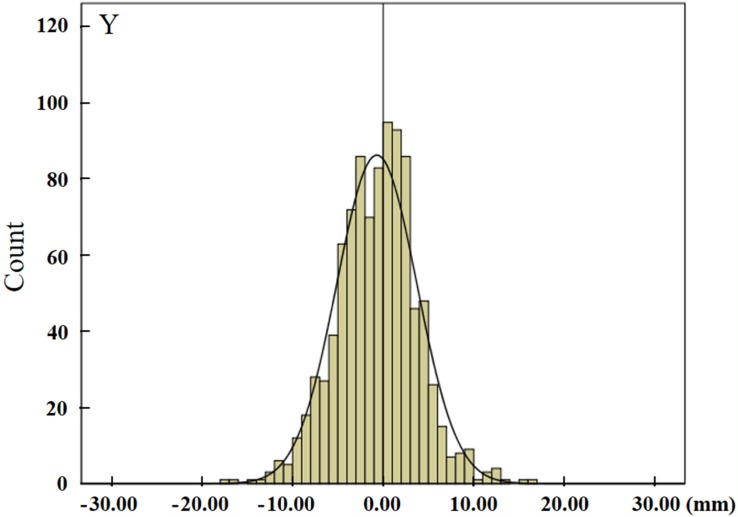

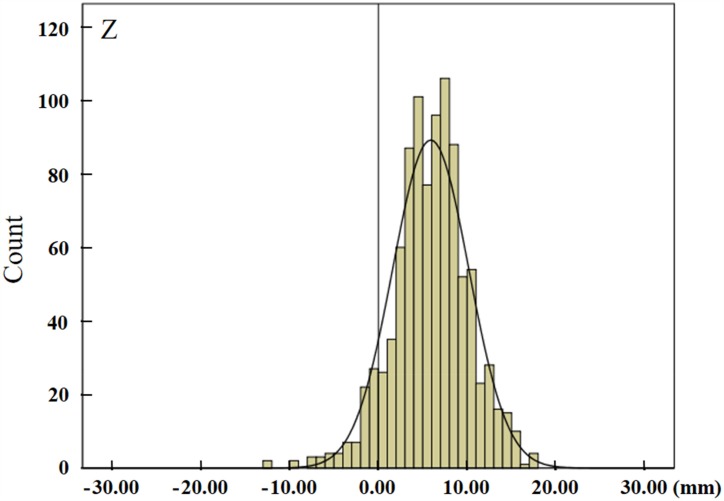

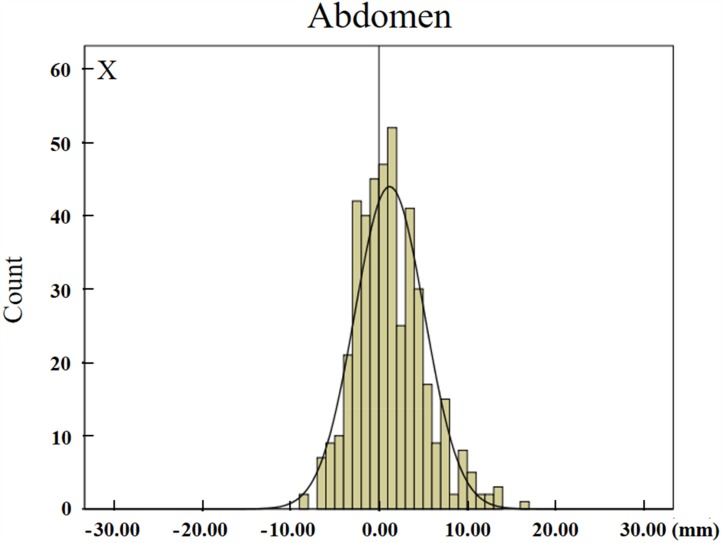

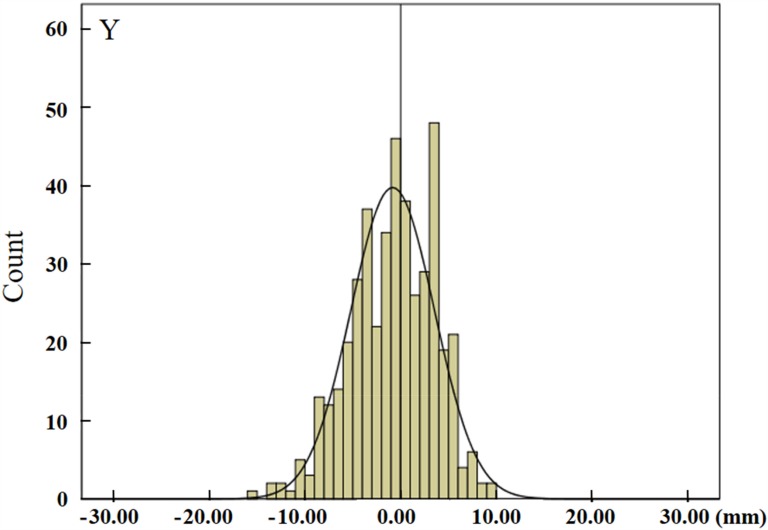

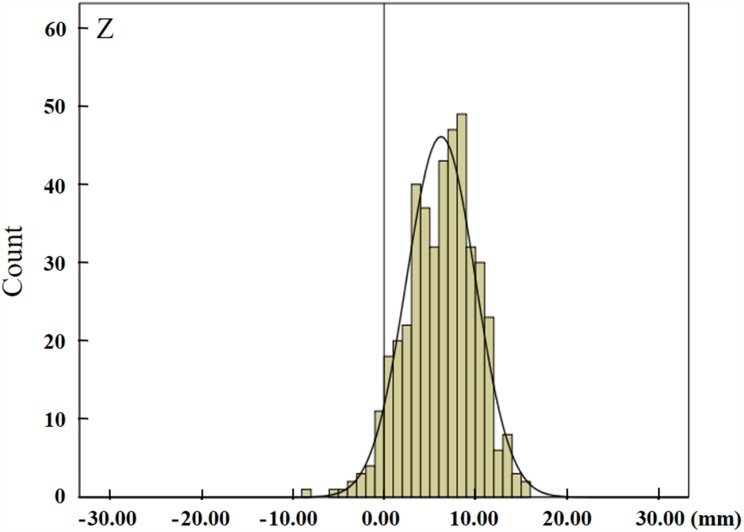

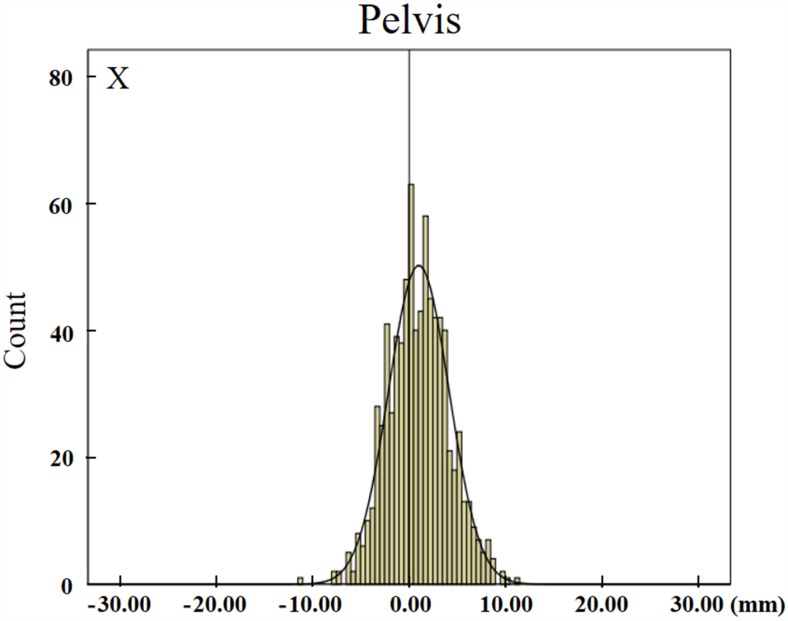

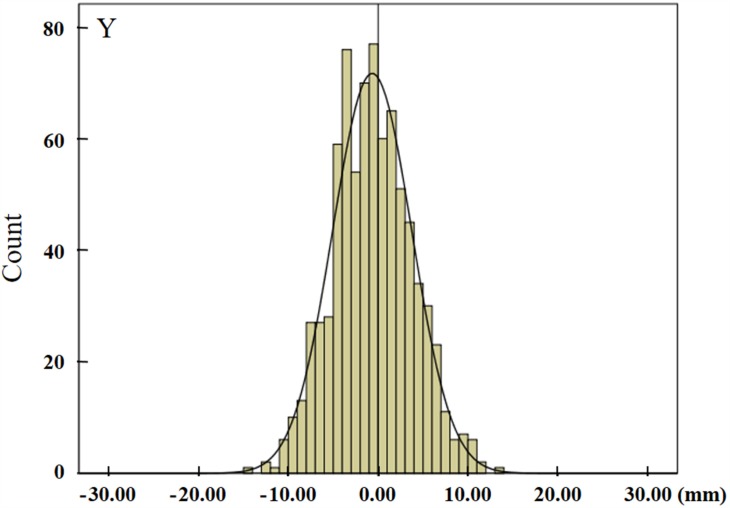

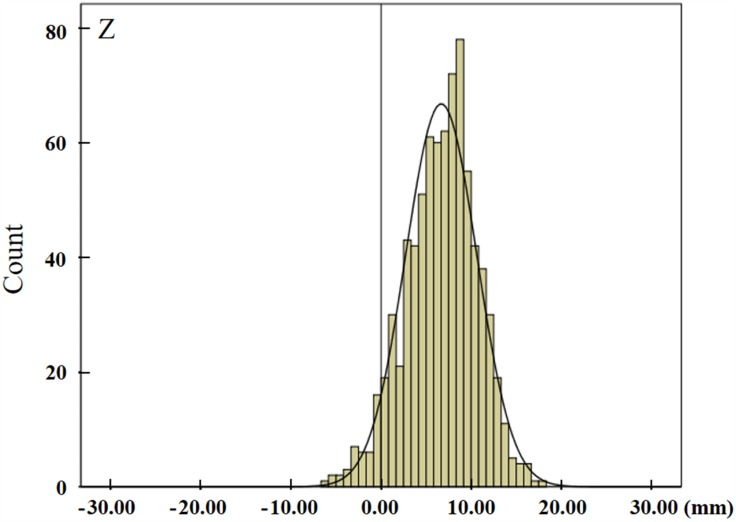
The distributions of translation setup errors along the X, Y, Z axes H&N means head and neck tumors. The setup errors along the X and Y axes were approximately symmetrical, however, the setup errors were not symmetrical along the Z axis.

The distributions of setup errors along the X, Y, Z axes in different tumor cases are shown in Figure 1. The setup errors along the X and Y axes were approximately symmetrical, however the setup errors were not symmetrical along the Z axis. In addition, the errors along the +Z direction were significantly higher than that along the-Z direction (*p*<0.05). The distribution of the setup errors on Z axes for head and neck tumors, thoracic tumors, abdominal tumors, and pelvic tumors were -0.8–6.9 mm, -2.5–14.3 mm, -1.1–13.6 mm, and -1.1–14.4 mm, respectively (Table 1).

**Table 1:** The 95% distribution range of setup errors in all tumors (mm)

	H&N		Chest		Abdomen		Pelvis	
	Lower	Upper	Lower	Upper	Lower	Upper	Lower	Upper
X	-4.2	4.8	-5.8	6.3	-6.5	8.9	-5.2	7.2
Y	-4.4	5.7	-9.4	8.0	-9.4	7.7	-9.3	8.0
Z	-0.8	6.9	-2.5	14.3	-1.1	13.6	-1.1	14.4

^*^H&N means head and neck tumors. The setup errors along the X and Y axes were approximately symmetrical, however, the setup errors were not symmetrical along the Z axis.

### The setup errors of different tumors

**Figure 2:**
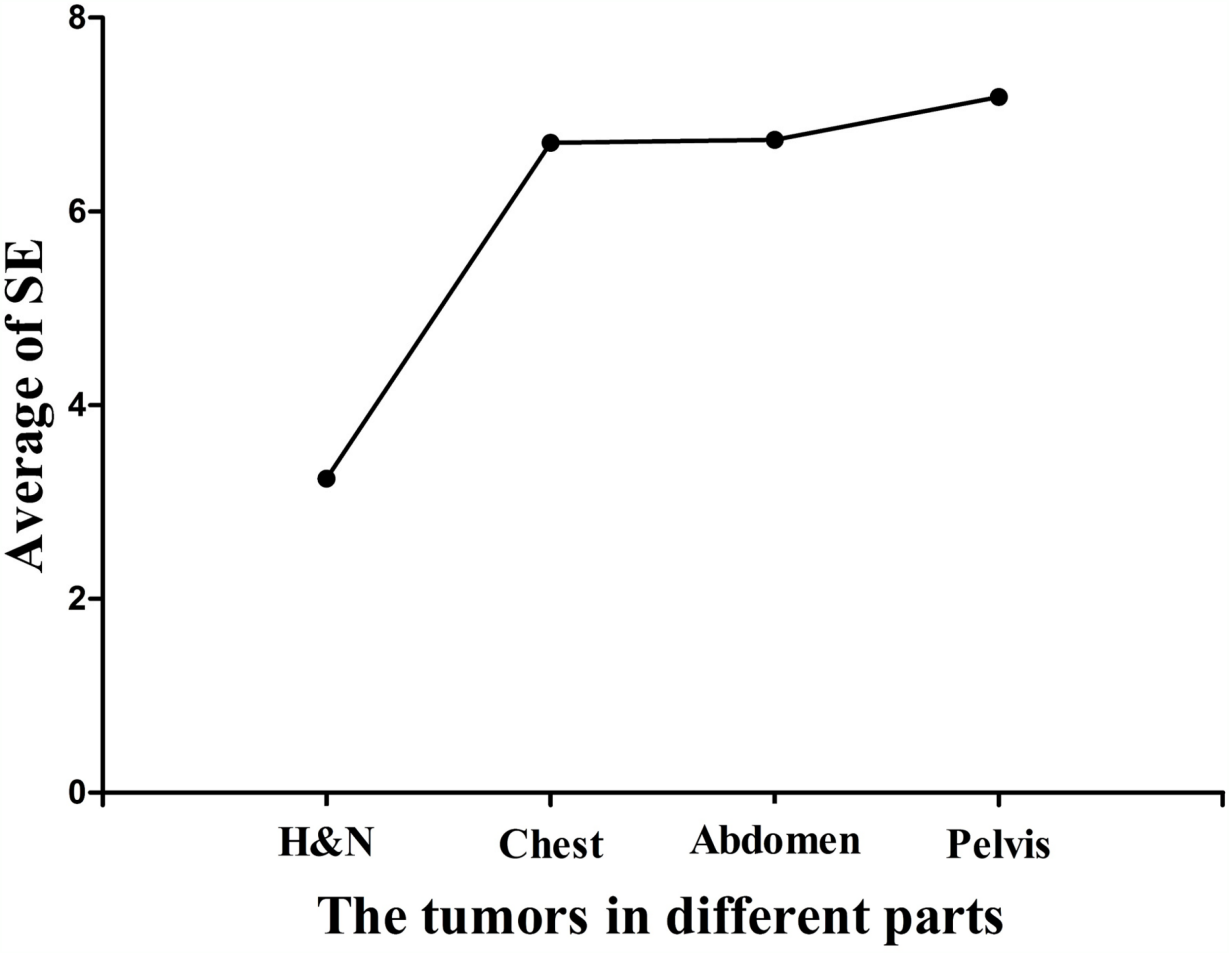
The average of setup errors along the +Z directions in all tumor cases H&N means head and neck tumors. It shows that the setup errors along the +Z direction increased proportionally to the distance to the head.

Table 2 shows the summary of the setup errors. These results demonstrated that the setup errors of head and neck tumors were smaller than other tumor cases along the six directions. The maximum setup errors among these cases were found along the +Z direction which were 1.9, 2.5, 3.5, and 3.7 times more than that along -Z direction in the head and neck tumors, thoracic tumors, abdominal tumors, and pelvic tumors, respectively. Figure 2 shows that the setup errors along the +Z direction increased proportionally to the distance to the head.

**Table 2:** The setup errors of tumors in different parts ($\bar{x}$ ± *s*) (mm)

	H&N	*P*	Chest	*P*	Abdomen	*P*	Pelvis	*P*
+X	1.61 ± 1.21	0.017	2.43 ± 1.88	0.699	3.67 ± 3.06	0.715	2.92 ± 2.13	0.020
-X	1.76 ± 2.11		2.55 ± 1.92		2.37 ± 1.77		2.17 ± 1.68	
+Y	2.26 ± 1.74	0.022	3.06 ± 2.64	0.376	3.18 ± 1.96	0.653	3.50 ± 2.61	0.081
-Y	1.83 ± 1.47		3.90 ± 2.91		3.98 ± 3.01		3.72 ± 2.66	
+Z	3.24 ± 1.76	0.000	6.71 ± 3.46	0.000	6.74 ± 3.25	0.000	7.18 ± 3.43	0.000
-Z	1.75 ± 1.61		2.64 ± 2.77		1.92 ± 2.00		1.92 ± 1.61	

^*^H&N means head and neck tumors. The setup errors of head and neck tumors were smaller than other tumor cases along the six directions. The maximum setup errors among these cases were found along the +Z direction. In addition, the errors along the +Z direction were significantly higher than that along the -Z direction (*P*<0.05).

### The margins for CTV to PTV

Systematic errors (Ʃ), random errors (σ) and CTV-PTV margins of all tumors are displayed in Table 3. The distributions range of systematic errors (Ʃ) were 0.8–1.4 mm, 1.0–2.7 mm, 0.9–2.4 mm, and 1.0–2.3 mm for head and neck tumors, thoracic tumors, abdominal tumors, and pelvic tumors, respectively. Random errors (σ) were smaller in head and neck tumors than other cases, and they were all less than 1.4 mm. The distributions range of random errors were 1.8–2.5 mm, 1.5–2.4 mm, and 1.3–2.8 mm for the thoracic tumors, abdominal tumors and pelvic tumors, respectively. The largest margins were found along -Z direction in all tumor cases; margins were 4.6 mm, 8.4 mm, 7.7 mm, and 7.7 mm for the head and neck tumors, thoracic tumors, abdominal tumors, and pelvic tumors, respectively.

**Table 3:** Systematic error (Ʃ), random error (σ) and CTV-to-PTV margin of tumors in different parts (mm)

	H&N			Chest			Abdomen			Pelvis		
	Ʃ	σ	Margins	Ʃ	σ	Margins	Ʃ	σ	Margins	Ʃ	σ	Margins
+X	0.8	1.1	4.3	1.0	1.8	3.7	2.1	1.9	3.5	1.3	1.8	3.7
-X	1.4	1.2	2.8	1.0	1.8	3.8	1.0	1.5	6.5	1.0	1.7	4.6
+Y	1.2	1.3	3.1	1.7	2.4	7.8	0.9	1.8	5.9	1.5	2.0	4.8
-Y	0.9	1.1	4.0	2.5	2.1	5.9	1.8	2.1	3.6	1.3	2.3	5.2
+Z	1.4	1.3	4.1	2.7	2.5	5.2	2.4	2.4	3.9	2.3	2.8	3.3
-Z	1.3	1.4	4.6	1.5	1.9	8.4	1.1	1.6	7.7	1.0	1.3	7.7

^*^H&N means head and neck tumors. Random errors(σ) were smaller in head and neck tumors than other cases, and the largest margins were found along -Z direction in all tumor cases.

Large differences in margins were observed along the ventral-dorsal directions, and the CTV-PTV margins along the-Z direction were 1.1, 1.6, 2.0, and 2.3 times more than that along the +Z direction in the head and neck tumors, thoracic tumors, abdominal tumors, and pelvic tumors, respectively.

## DISCUSSION

The CTV-PTV margins of different tumors showed heterogeneity when patients underwent helical tomotherapy. The heterogeneity was more significantly different along the Z axis, which meant that using previous methods to calculate CTV-PTV margins along the three directions could result in uncertainties. These uncertainties may increase the risk of target missing and the radiation exposure of OARs.

The distributions of setup errors (Figure 1) demonstrate that setup errors for head and neck tumors were smaller than for other tumors. Among all tumor cases, head and neck tumors had the smallest random errors (σ), thus indicating the repeatability of the setup was best in head and neck tumors.

Note that these results were consistent to previously published articles; for instance, Schubert et al showed that the distributions for brain, head, and neck treatments were narrow and sharp compared to lung and prostate tumors, and they also showed that the systematic errors and random errors were smaller in all translational directions for brain, head, and neck treatments [19]. One possible explanation for the setup differences between the various treatment sites could be the use of different immobilization methods. The thermoplastic head masks were used for head and neck immobilization, which could limit the movements of patients effectively, thus, result in fewer setup errors. On the other hand, the setup errors of the thoracic tumors, abdominal tumors, and pelvic tumors were larger, which may be due to respiration, immobilization approaches used, and couch sag.

Previous studies demonstrated that the largest systematic errors (Ʃ) were found along the Z axis in head and neck tumors, prostate tumors, and other tumors [20-22]. Our results were consistent with previous findings, showing that the largest systematic errors (Ʃ) were in the +Z direction. In addition, these results may be associated with the effects of couch sag by analyzing the setup errors of all tumor cases. Since the patients were positioned to the setup lasers outside the bore, and treatment was conducted inside the bore, the positioning may not take the absolute tomotherapy couch sag into account [19]. Since the couch lacks the support from the tabletop, it could increase the setup errors along the +Z direction. The average of setup errors for the head and neck tumors, thoracic tumors, abdominal tumors, and pelvic tumors along the +Z direction reached 3.24 mm, 6.71 mm, 6.74 mm, and 7.18 mm, respectively (Figure 2).

These results showed that the setup errors were increased with the shift of treatment sites from the head and neck to pelvis, which might be associated with the distance from the tabletop which may result in a lower center of gravity. Thus, the CTV-PTV margins among Z axes would increase with the couch sag.

The average of setup errors for all tumors were compared along different directions in the same axes; the setup errors between +Z and –Z were significantly different in all tumors (*p*<0.05). These results indicate that the heterogeneity of setup errors in ventral-dorsal directions for all parts of tumors should be considered when designing a treatment plan.

The reduction of couch sag influences and solving the heterogeneity of setup errors are issues that need to be resolved. One possible solution could be adjusting the couch sag when the patients undergo tomotherapy for the first time. However, the shortcoming of this method can be intra-subject changes over time, such as patients’ possible changing weight. Another possible solution could be adjusting the couch sag before patients undergo tomotherapy treatment every time. However, the efficiency of the treatment could be compromised because of the time spent. Future studies may formulate the optimal methods in calculating the CTV-PTV margins before patients accepted tomotherapy. Such optimal methods could reduce the time spent in modifying and verifying the treatment plan caused by inadequate estimation of setup errors.

In this study, the CTV-PTV margins were calculated according to van Herk’s formula, which can derive a margin with an accuracy of 90% such that the patients receive a minimum cumulative CTV dose of at least 95% of the prescribed dose [18]. The dose gradients of tomotherapy were clearer between tumors and surrounding normal structure, and were more sensitive to the size of CTV-PTV margins. There could be a dose loss in the target or a dose increase in organs at risks if the margins were not appropriate.

**Figure 3:**
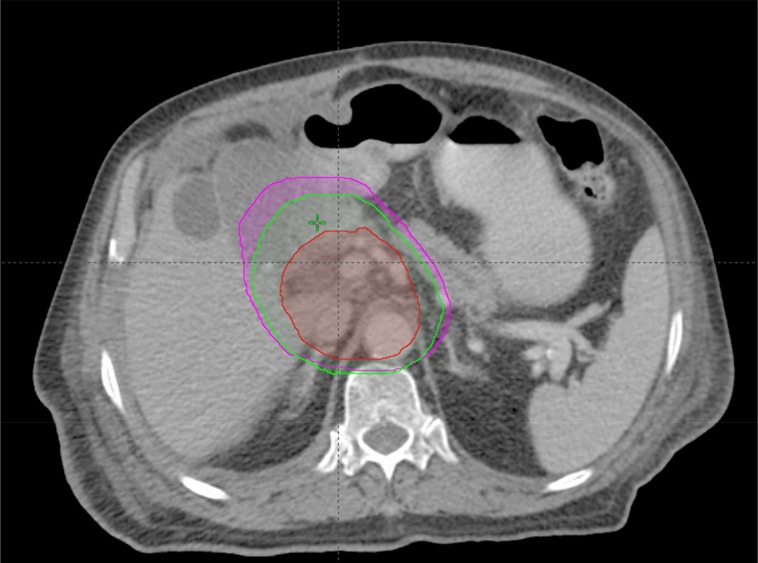

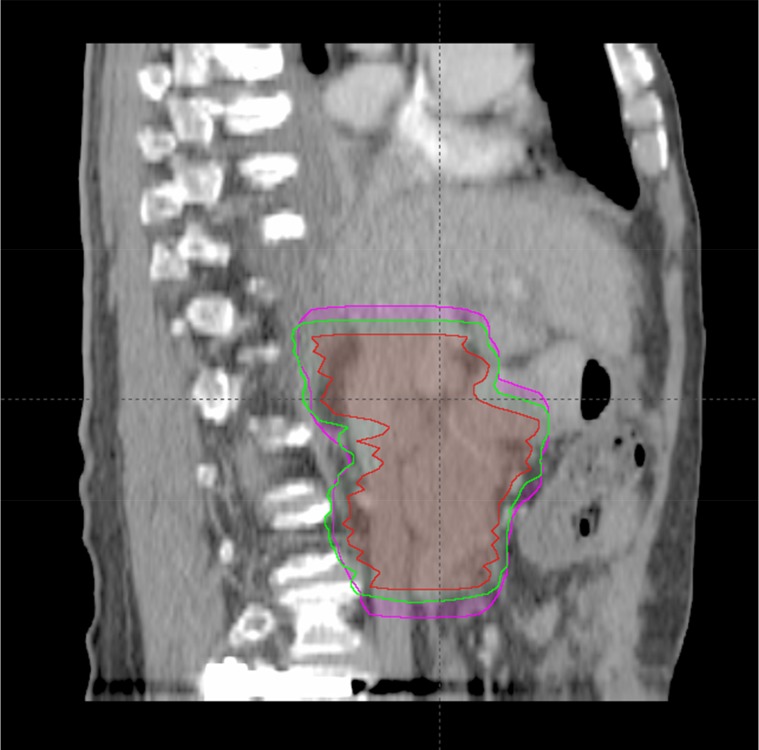

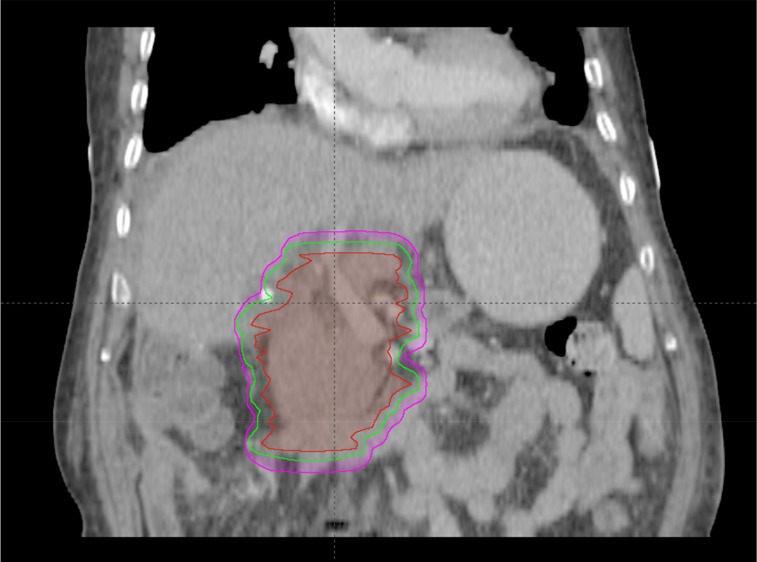

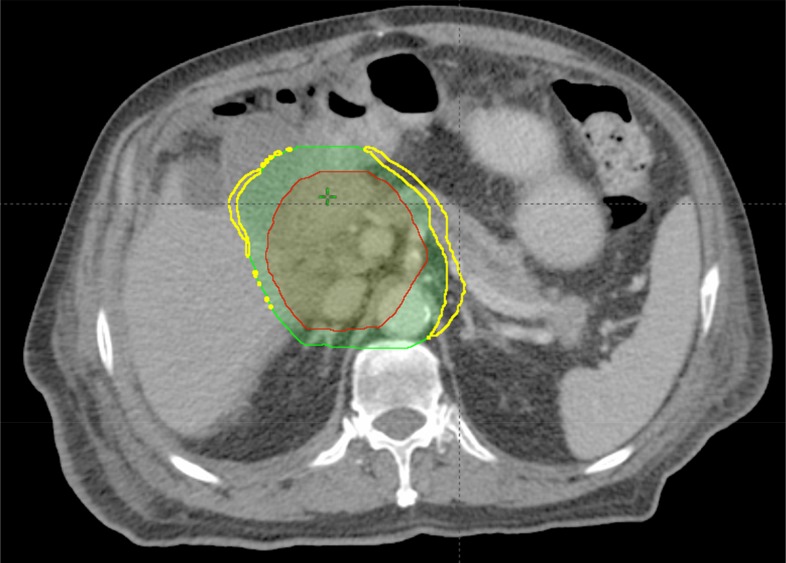

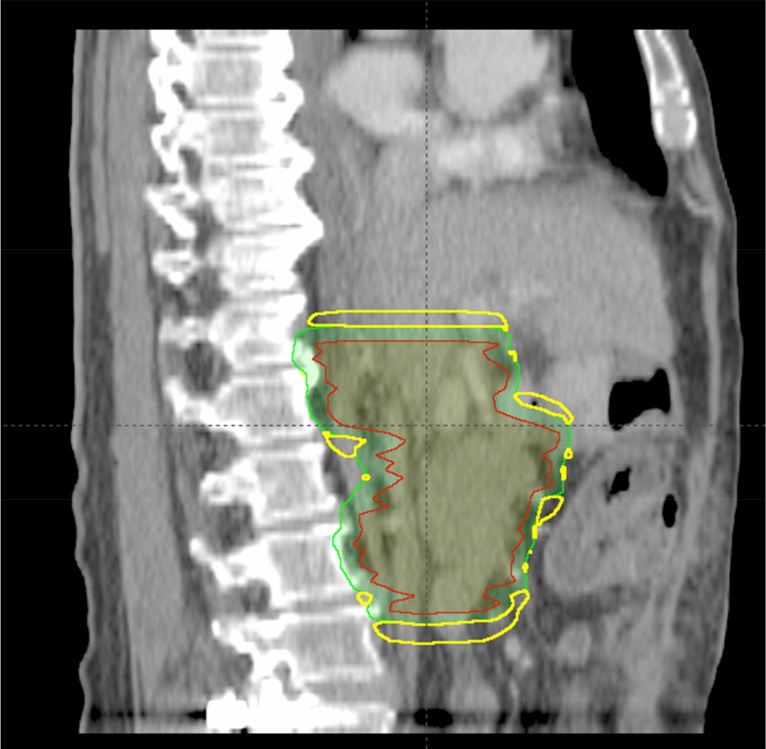

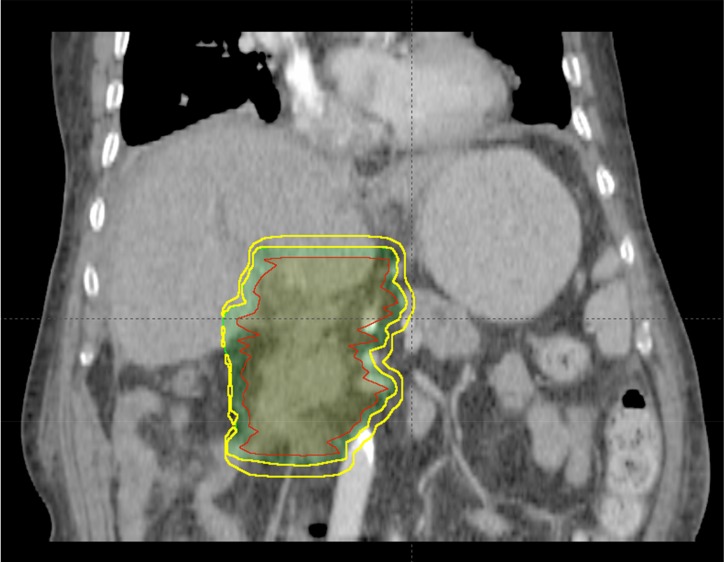

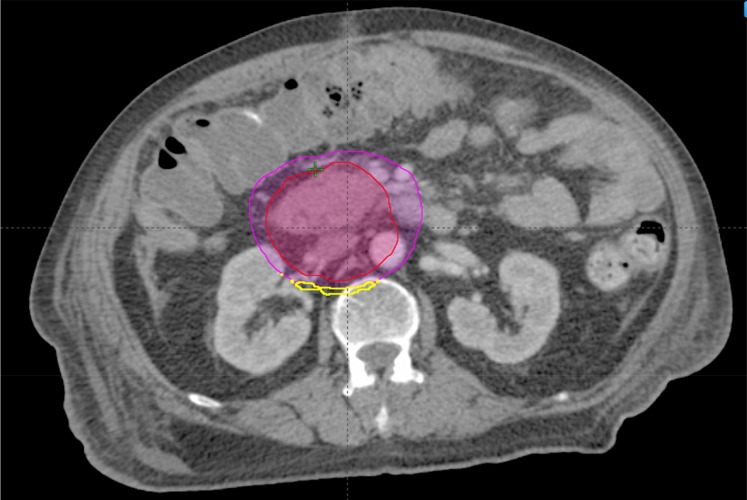

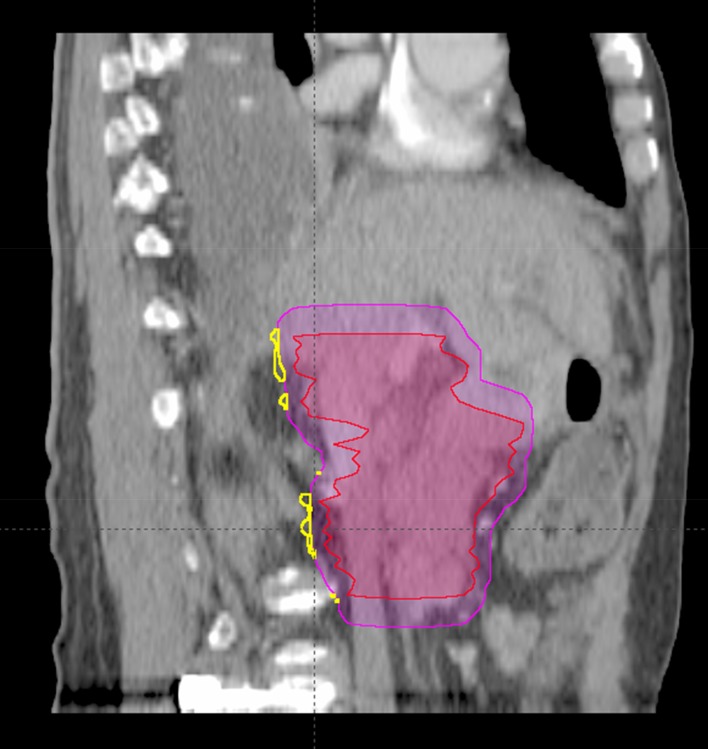

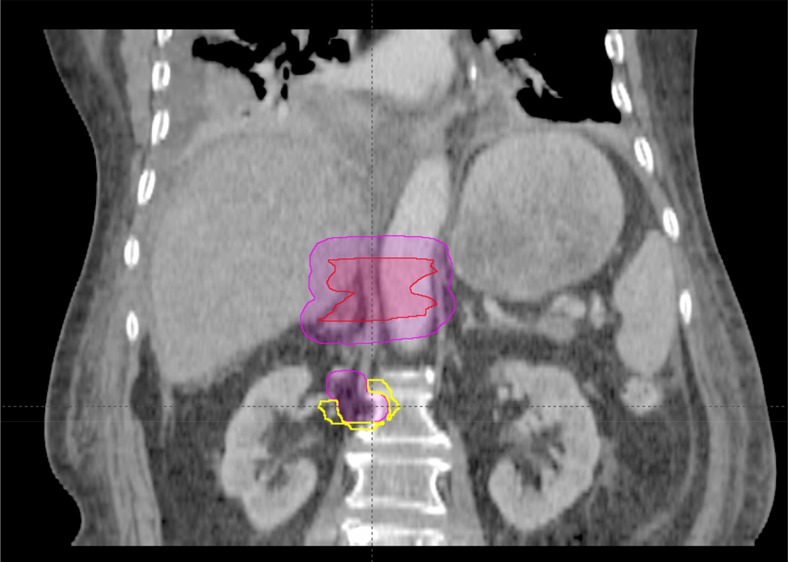
An example for abdomen tumors **(a)** CTV (in red) was 416.08 ml, PTV_1_(in purple; expended margins of Zhou’s recommendation) was 839.18 ml; the PTV_2_(in green; expended margins of this study) was 691.35 ml. **(b** and **c)** The relationship of three volume, the intersectional volume of PTV_1_ and PTV_2_ was 682.89 ml, and $V_{PTV_{1}-PTV_{2}}=$140.16 ml (b), $V_{PTV_{2}-PTV_{1}}=$4.45 ml (c).

Our results demonstrated a great difference between ventral and dorsal directions for thoracic tumors, abdominal tumors, and pelvic tumors. The CTV-PTV margins along the ventral direction were 5.2 mm, 3.9 mm, and 3.3 mm for thoracic tumors, abdominal tumors, and pelvic tumors, respectively; while the CTV-PTV margins along the dorsal direction were 8.4 mm, 7.7 mm, and 7.7 mm for thoracic tumors, abdominal tumors, and pelvic tumors, respectively. Note that the mean values of these results were consistent with the study by Zhou et al which proposed that the margins in lung, abdomen, and prostate cancers were 6.8 mm, 4.1 mm, and 5.4 mm, respectively, along the Z axis [23]. However, if one expended the CTV to PTV by using these margins, the margin would be larger and smaller along the +Z and-Z directions, respectively (Figure 3), and this would lead to OARs dose increase or tumor dose reduction. Such an argument could also apply to abdominal and pelvic tumors. Thus, it might be important to expand the heterogeneous margins along all directions for these tumors.

This study suggests expanding the heterogeneous CTV-PTV margins when patients undergo tomotherapy treatment. Our results demonstrated that the safety of radiotherapy could be improved without MVCT scans by using these CTV-PTV margins. In addition, the potential insufficient irradiation of tumors or the potential excessive exposure of an OAR caused by inadequate or excessive CTV-PTV margins could be avoided. However, dose metric superiority was not conducted in this study. In addition, other factors that affect setup errors and CTV-PTV margins were not considered, such as the external fiducial type and placement location, the positioning laser accuracy, as well as the tumor delineation uncertainty of the CTV [10, 19].

This study thought that the CTV-PTV margins for patients with different types of tumors had heterogeneity during tomotherapy. Future studies should formulate the optimal methods in calculating the CTV-PTV margins before patients accepted tomotherapy using information along the left, right, head, foot, ventral, and dorsal directions. Such optimal methods could reduce the time spent in modifying and verifying the treatment plan caused by inadequate estimation of setup errors.

## MATERIALS AND METHODS

### Patients

Positioning corrections were analyzed for 151 patients (92 male and 59 female; 56 ± 15 years old) receiving helical tomotherapy treatment between September 2015 and May 2016 at Shandong Cancer Hospital Affiliated to Shandong University (Jinan, China). Among the 151 patients, 53 patients had head and neck tumors, 45 patients had thoracic tumors, 20 patients had abdominal tumors, and 33 patients had pelvic tumors. The patients received 1094 MVCT scans, 960 MVCT scans, 435 MVCT scans, and 792 MVCT scans, respectively. This study was approved by the Research Ethics Board of the Shandong Cancer Hospital, and informed consent was obtained from all patients.

### MVCT acquisition

Head and neck patients were immobilized with thermoplastic head masks whereas thoracic patients were immobilized with thermoplastic masks and vacuum pads. Abdominal and pelvis patients were immobilized with the vacuum pads. All patients were placed in the supine position, and received radiation therapy with head-first. All CT images were acquired with a Philips Brilliance CT Big Bore scanner (Phillips Medical Systems, 96 Highland Heights, OH, USA). The slice thickness was 3 mm. The CT images were subsequently transferred to the treatment planning system for treatment plan designs. Helical MVCT images were acquired at every treatment fraction after the patient was immobilized and positioned with localization lasers. The slice thickness was 4 mm.

### Image registration and setup errors calculation

MVCT images were registered to simulation CT images. A radiation therapist subsequently checked the settings before each treatment. Image registration was based on bony anatomy for some tumors in bony structures such as head and neck tumors, while image registration was based on bony and soft tissue anatomy for the tumors in soft tissue such as breast tumors. Image registration was first performed using an automatic process and followed by manually checking. Adjustments were applied if necessary before treatment. Positioning corrections were applied by couch shifts in X (left-right), Y (head-foot) and Z (ventral-dorsal) axes. In addition, the setup errors on these directions were recorded.

### Setup errors and margins calculation

The distributions of the setup errors in different tumor cases were plotted, and the 95% distribution range of the setup errors was defined as mean ± 1.96 × standard deviation. The setup errors of different tumor cases along the +X (left),-X (right), +Y (head), -Y (foot), +Z (ventral), and-Z (dorsal) axes were measured and displayed as mean ± standard deviation ($\bar{x}$ ± *s*).

Systematic errors (Ʃ) and random errors(σ) of different tumor cases along the six directions were analyzed. The systematic errors (Ʃ) were computed by taking the standard deviation of the mean of displacements in each individual patient over a population in different tumor cases, which was stochastic in nature for a group of patients. For instance, one patient error might be due to postoperative edema and another might be due to wrong positioning before treatment. The random errors (σ) were defined as the root mean square of the random error distribution, which were computed from the standard deviations of individual patients over a population in different tumor cases. Random deviation occurred due to day-to-day variations in patient setup. Lastly, the CTV-PTV margins of different tumor cases were calculated by van Herk’s formula: M = 2.5 Σ+0.7 σ [18].

### Statistical analysis

All data was analyzed using SPSS v19.0 software (SPSS Inc., Chicago, IL). Parametric tests were applied to data with normal distribution and homogeneity in variance, while non-parametric tests were applied to data without them. In particular, independent sample t-test was selected as the parametric test, while Mann–Whitney U test was selected as the non-parametric test. The differences were considered as statistically significant when *p<*0.05.

## Abbreviations

CT: computed tomography
MVCT: Megavoltage computed tomography
IMRT: intensity modulated radiation therapy
IGRT: image guide radiation therapy
ART: adaptive radiation therapy
OAR: organs at risks
CSI: craniospinal irradiation
CTV: clinical target volume
PTV: planning target volume
